# Hybrid-Type Transparent Organic Light Emitting Diode with High Contrast Using Switchable Windows

**DOI:** 10.3390/ijms24021097

**Published:** 2023-01-06

**Authors:** Seongwook Choi, Chang-Hee Lee, Ju-Hyeok Choi, Sung-Hoon Choi, Bongsoon Kang, Gi-Dong Lee

**Affiliations:** 1 Department of Electronics Engineering, Dong-A University, Busan 49315, Republic of Korea; 2 LG Display Co., Ltd., Gumi 39394, Republic of Korea

**Keywords:** OLED, transparent display, liquid crystal window, high contrast, polarized mode, non-polarized mode

## Abstract

Transparent organic light emitting diode (OLED) display is one of the most promising devices among next-generation information displays because of beneficial characteristics, such as self-emissive and optically clear properties. Nevertheless, in conventional transparent OLED display devices, there are serious intrinsic problems in terms of the transmittance in the dark state because of empty windows in the cell, so the contrast ratio of the transparent OLED display would be deteriorated even though it can exhibit excellent bright state. In general, the transparent mode using the OLED device applies an empty area in each pixel because an emitting device could never reveal the background image, so the transparent OLED should contain the empty area in the pixel for transparent images. This may cause the optical degradation in the dark state. To solve this problem, we propose hybrid-type transparent OLED display modes that apply a liquid crystal (LC) to the transparent window part of the empty space. In this paper, we applied two dichroic LC modes— which use an electrically controlled birefringence (ECB) mode (Heilmeier type) for the polarized mode and a cholesteric LC mode (Guest-Host mode) for the non-polarized mode—to the empty area. In each hybrid mode, we have observed optical performance, including the transmittance in the dark/bright state, contrast ratio and response time as a function of cell parameters. As a result, we confirmed that the dark state and the contrast ratio could be improved by applying the proposed modes without serious decay of the transmittance in the bright state.

## 1. Introduction

Transparent displays are receiving considerable attention because they can be harmonized naturally without seeming disconnected from the surroundings and can be used anywhere where glass windows are installed [1,2,3,4]. Unlike conventional displays, such as smart phones, tablets and TV, information can be displayed on a simple transparent glass, which is used as a useful function in windows, head-up displays and even buildings [5,6,7]. In addition, transparent displays can be used in augmented reality, which can be used in navigation and advertising by enabling interaction between displayed information and environmental backgrounds.

So far, many studies have been reported on applying various technologies to transparent displays, mainly transparent displays using LCD and transparent displays using OLED devices [8,9]. In the case of a transparent LCD, it can be easily made transparent with full color [10]. However, the transmittance becomes low because of the built-in polarizer, color filter and optical correction film (<14%). Many researchers have attempted to improve the transmittance of transparent LCDs, such as field sequential color technology [3]. However, the transmittance achieved is only 16%. In addition, due to large size, weight and poor color gamut properties, it still needs improvement for practical use.

The transparent OLED is a promising candidate for transparent display applications with lightweight and good optical properties [11,12,13]. The transmittance of transparent displays without polarizers can be increased up to 54% and is still improving by developing transparent electronic devices and organic materials [14]. Furthermore, this self-luminous device without an external light source not only maintains good image quality but also enables the development of lightweight and portable transparent devices, which can be used efficiently in augmented reality. However, in transparent OLED display devices, there is a serious intrinsic problem in terms of transmittance in a dark state. In general, transparent modes using OLED devices apply empty areas in pixels because light emitting devices can never reveal background images, so transparent OLEDs must include empty areas in pixels for transparent images. Even if the transparent OLED display device shows excellent transmittance, the transmittance in the dark state is greatly deteriorated due to the transmittance of the empty space, and thus the contrast ratio is reduced.

For the improvement of the dark state of the transparent OLED cell, several attempts were made, in the previous papers [15,16], to block the empty area using a LC switching cell. The papers have shown an improved optical performance compared to the conventional transparent OLED cell. However, the structure of the most previous papers applied to a stacked panel consists of an OLED cell and a cholesteric LC cell, so the cell thickness of the transparent OLED becomes higher. Furthermore, transmittance of the window part in the bright state becomes lower due to the stack structure consisting of the cholesteric LC cell and two empty glasses.

In this paper, we propose a hybrid-type transparent display that combines an OLED device and LC mode cell to overcome drawbacks [17]. The transparent window of the empty area is replaced by a LC mode cell to develop a high-performance transparent display. In general, the OLED device applies a polarization sheet to the surface of the device to get rid of reflected light on the glass surface, which deteriorates visibility of the light from the OLED. In the case of the transparent OLED mode, however, the polarizer sometimes is not applied for high transmittance display. Therefore, we consider two transparent OLED modes for a polarized mode and a non-polarized mode by applying two LC modes to the transparent empty window. The first is an ECB mode LC cell using Heilmeier type dichroic dyes for the polarized mode, and the second is a cholesteric LC mode using Guest-Host mode for the non-polarized mode. This paper contains the detailed transparent OLED structures for both the polarized mode and the non-polarized mode and shows the optical improvement in terms of contrast and transmittance through experiments.

## 2. Result and Discussion

### 2.1. Proposed Optical Structures of the Hybrid-Type Transparent Display

**Figure 1:**
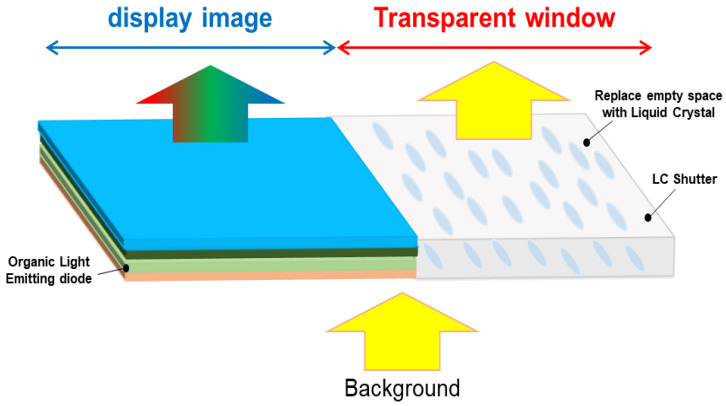
The conceptual diagram of the proposed hybrid-type transparent OLED display.

Figure 1 shows the concept of the proposed hybrid-type transparent display mode using the OLED device. Basically, the conventional transparent display, using an OLED device, consists of an information display part using an OLED cell and a transparent part using an empty area. To improve the light leakage in the dark state due to the empty area, we substitute the empty area with switchable windows for achieving both the good dark state and the high transmitted bright state together. This concept can be realized by replacing the empty space with LC switching cell, which can make the dark state characteristics better and improve the contrast ratio [18,19,20].

#### 2.1.1. Proposed Switching Window Using a Heilmeier Type LC Cell for Polarized Mode Transparent OLED Cell

In the transparent window part, an ECB mode LC cell using a Heilmeier type dichroic dye is used to control light absorption on specific wavelengths by adding dichroic dyes for the polarized mode. Dichroic dyes are rod-shaped anisotropic molecules, such as LC, in which the absorption of light varies depending on the polarization direction. The dichroic dye can be easily aligned along the alignment direction of the LC molecules [21,22]. Because of dichroism, dye molecules strongly absorb incident light polarized parallel to the absorption axis. The transmittance of LC cells using dichroic dyes depends on the alignment of dye molecules, the absorption coefficient of dye, cell gap of LC and dye concentration. By increasing the cell gap or dye concentration, the transmittance in the bright state may be reduced; device contrast can instead be increased due to reduction of the light transmittance in the dark state. The response time and driving voltage of the LC cells with the dichroic dye depend on the cell gap and dye concentration, and the solubility of the dye molecule may be problematic. Therefore, optical dependency of the transparent OLED cell with the proposed structure on the cell parameters should be studied for desired optical performance.

**Figure 2:**
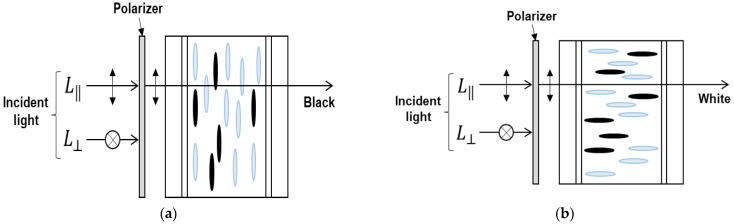
A switching window for the polarized mode transparent OLED cell using a dye doped LC mode with the Heilmeier structure: (**a**) Dark state and (**b**) Bright state.

Figure 2 shows the driving concept of the proposed LC mode for the polarized mode. Basically, we use a single polarizer in front of the LC cell so that emerging light passing through the device contains polarization information. In Figure 2, the polarizer makes the light pass through the cell with the parallel polarizing state so that the device removes the perpendicularly polarized light in the device operation. In Figure 2a, the dichroic dye used in the LC strongly absorbs the incident polarization component L‖, which is parallel to the molecular axis, representing a black state as light is transmitted through the LC cell. Figure 2b shows the transparent state of the transparent cell. In this state, the orientation of the LC molecule is switched perpendicular to the incident light by the voltage applied from the outside, and thus, absorption of the light is decreased and becomes transparent state. When a voltage is applied to the mixture of the dichroic dye and the LC, the orientation direction of the molecule changes so that the light absorption characteristics change, and as a result, switching black and transparent becomes possible. 

Depending on the mixing ratio of dichroic dyes in the LC and the thickness of the LC layer, the optical characteristics, such as contrast ratio, response time and driving voltage, are greatly affected. Therefore, in order to implement the high-quality image of the polarized mode, showing high optical characteristics as a light control device, we have analyzed the characteristics according to the mixing ratio of dichroic dyes and LC cell gap. By comparing this, transparent OLED devices of the polarized mode were designed and optimized according to the optimized conditions.

#### 2.1.2. Proposed Switching Window Using a Ch-LC Cell with Dichroic Dye for Non-Polarized Mode Transparent OLED Cell

**Figure 3:**
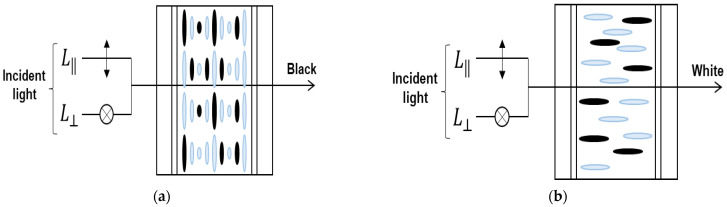
A switching window for the non-polarized mode transparent OLED cell using a Ch-LC layer with the dichroic dye: (**a**) Dark state and (**b**) Bright state.

In the case of the non-polarized mode, the Cholesteric LC (Ch-LC) cell using GH effect, which is a mode of controlling light absorption by adding dichroic dyes absorbing light of a specific wavelength, was used in the transparent window part. As shown in Figure 3, a cholesteric liquid crystal or chiral nematic liquid crystal is a LC added with a chiral dopant that induces a periodic helical structure to the nematic LC and has a helical structFure arranged by twisting the direction of the nematic LC along the helical axis. The helical direction of the Ch-LC is determined by the chiral unit, which is called the helical pitch, and the helical axis acts as an optical axis. In Figure 3, the molecules of the dichroic dye spread out to all directions along LC molecular direction so that the light passing through the cell can be absorbed in all directions. Therefore, this structure does not require the use of polarizers for polarization filtering, and the optical performance of the device can be varied depending on how we control the helical pitch of the LC cell, the cell gap and the absorption properties of the dye. In this paper, we designed the non-polarized mode as a light control device for the transparent window part having such properties by adding dichroic dyes [23,24,25].

### 2.2. Measured Optical Performances of the Polarized Mode and the Non-Polarized Mode for Hybrid-Type Transparent OLED Display

#### 2.2.1. Electro-Optical Characteristics of the OLED Cell as a Display Area

**Figure 4:**
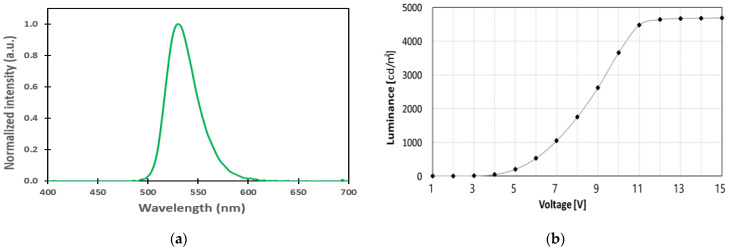
Measured electro-optical characteristics of the OLED cell: (**a**) electroluminescence spectrum and (**b**) luminance on voltage characteristic.

The electro-optical characteristics of the OLED used in the proposed transparent OLED display are shown in Figure 4. The peak wavelength of the OLED used is 530 nm, and the full width at half maximum (FWHM) is 34 nm, as shown in Figure 4a. The dependence of luminance on voltage is depicted in Figure 4b. The device turns on at 5 V and reaches maximal luminance of 4644 cd/m^2^ in front of the substrate and 2162 cd/m^2^ in front of the polarizer on the substrate at 12 V. In this study, in order to calculate the electro-optical characteristics of the polarized mode and non-polarized mode for proposed transparent OLED device, the OLED was driven at 12 V, which emits light with maximum efficiency.

#### 2.2.2. Proposed Switching Window Using a Heilmeier Type LC Cell for Polarized Mode Transparent OLED Cell

Before we measure the light luminance of the window part of the proposed transparent OLED cell, we have measured light luminance of the window part of the conventional transparent cell. As we mentioned above, the conventional transparent cell applies an empty cell for the transparent part. For the comparison and estimation of the proposed transparent cell, we fixed the light luminance of the backlight at 1000 cd/m^2^.

**Figure 5:**
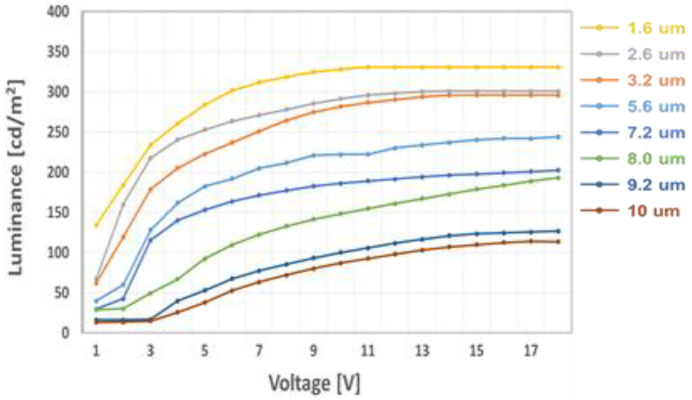
Measured voltage-luminance of the polarized mode LC cell as a parameter of cell gap at 6.2 wt % of the dichroic dye concentration.

**Figure 6:**
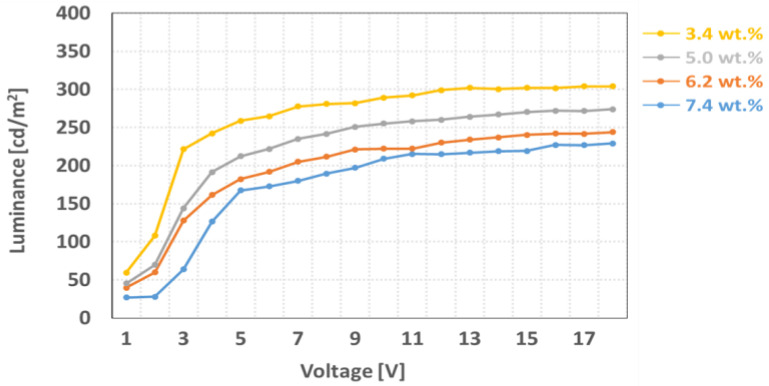
Measured voltage-luminance of the polarized mode LC cell as a parameter of the dye concentration at 5.6 µm of the cell gap.

Figure 5 shows that the dependency of voltage-transmittance curve depends on the thickness of LC layer. We have varied the cell gap of the LC cell from 1.6 µm to 10 µm with the same dye concentration value of 6.2 wt %. In Figure 5, we observed the light intensity in the dark state decreased to 13 cd/m^2^ from 112 cd/m^2^ and in the bright state decreased to 120 cd/m^2^ from 331 cd/m^2^ by increasing the thickness of the LC layer. This is because the liquid crystal with injected black dyes increases as the cell gap increases. Due to the low chiral twist power of the LC cell, the value of ∆V (V_on_/V_off_) is relatively high [26]. Detailed measurement data are shown in Table 1. Similarly, we have also measured the dependency of the voltage-transmittance curve, depending on the dye concentration in the LC layer as shown in Figure 6 and data are shown in Table 2. For the experiment, the dye concentration in the LC layer has changed from 3.4 wt % to 7.4 wt % with the LC layer of 5.6 µm. When observed, the light intensity in the dark state decreased to 29 cd/m^2^ from 59 cd/m^2^ and in the bright state decreased to 229 cd/m^2^ from 304 cd/m^2^ by increasing the dye concentration. Due to the high viscosity of the dichroic dye, we can also observe the slightly increased response time by increasing the dye concentration, even though we applied the same thickness of the LC layer.

#### 2.2.3. Electro-Optical Characteristics of the Non-Polarized Mode LC Cell as a Window Part

**Figure 7:**
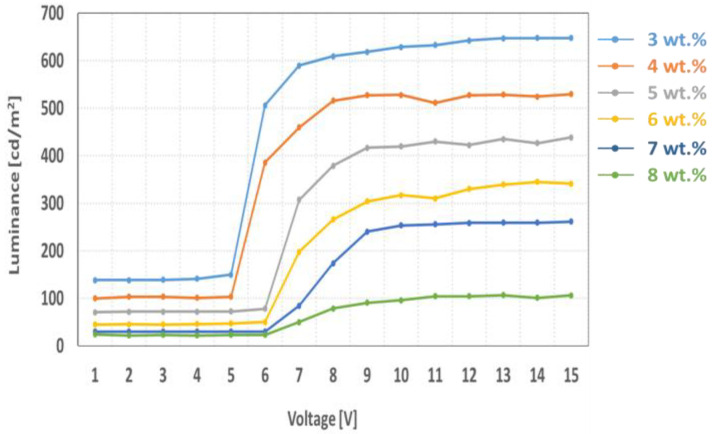
Measured voltage-luminance of the non-polarized mode LC cell as a parameter of the dye concentration at 10.0 µm of the cell gap.

Figure 7 and Table 3 show the dependency of optical characteristics in transparent and dark states of the non-polarized mode transparent OLED cell on the dichroic dye concentration. For uniform distribution of the dye molecules, we have fabricated 2π-twist Ch-LC cell with the LC thickness of 10 μm. The dye concentration in the LC layer has changed from 3.0 wt % to 8.0 wt %. In Table 3, the measured light transmittance in the dark state decreased to 18 cd/m^2^ from 138 cd/m^2^, and in the bright state, decreased to 110 cd/m^2^ from 654 cd/m^2^ by increasing the dye concentration. Compared to the polarized mode LC cell that applies the non-twisted LC layer, the non-polarized mode LC cell shows a small value ∆V because of 2π-twist LC layer. We can also see that response time of the non-polarized mode is higher than the polarized mode because of the high chiral twist power of the LC layer.

### 2.3. Comparison of Contrast Ratio for the Proposed Two Transparent OLED Device

With the measured optical performances of the OLED area and the window area, we can compare the optical characteristics of two transparent modes through experiments. In our experiment, we could simply measure the light luminance of the conventional transparent OLED cell at on/off state with 5650 cd/m^2^ and 1000 cd/m^2^, so that the calculated contrast ratio was 5.6:1. Table 4 and Table 5 show the calculated contrast ratio of the transparent OLED cell applying the polarized mode LC window. The measured light luminance of the OLED area was 2162 cd/m^2^ that was decreased to almost half-scale because of the polarizer. Instead, the measured light luminance in the dark state can be decreased to 13 cd/m^2^ at 10 µm of the LC cell gap and 6.2 wt % of the dye concentration. Therefore, we can expect high contrast ratio due to the polarizer even though the light luminance in the bright state deteriorates. Table 6 shows the calculated contrast ratio of the transparent OLED cell applying the non-polarized mode LC window with the cell gap of 10 µm. In this mode, the light luminance in the bright state could be increased to 4644 cd/m^2^ because of the non-polarizer state, despite relatively high luminance in the dark state compared to the polarized mode cell. Obviously, we can observe the trade-off between the polarized mode and the non-polarized mode transparent OLED cell on the optical luminance in the bright state and the contrast ratio. For example, under the condition of 6.2 wt % of dye condition and 10 μm of the LC layer thickness, the polarized mode shows low luminance in the dark state (≈13 cd/m^2^) compared to the non-polarized mode (≈45 cd/m^2^). As for the bright state, obviously the non-polarized mode can show the high transmittance (≈5000 cd/m^2^) compared to the polarized mode (≈2280 cd/m^2^) and low ∆V to achieve the high brightness. Therefore, the contrast ratio of the polarized mode (175:1) is higher than the non-polarized mode (111:1) due to the dark state, and this shows high improvement compared to the conventional transparent OLED without a serious decay of the light luminance in the bright state.

## 3. Methods and Materials

The ECB mode LC cell, using Heilmeier type dichroic dyes for the polarized mode and Ch-LC cell for the non-polarized mode, was fabricated as a single sample, and the electro-optical characteristics were analyzed. In order to fabricate the two cases, the ratio of the dichroic dyes was controlled 3–8 weight percent (wt %), and the cell gap of LC was controlled to 1.6–10 µm by ball spacer. Dichroic dyes have been developed to make black dyes for black and transparent OLED display shutters. 

Black dyes were fabricated by four types of dyes (Nematel Gmbh & Co., Mainz, Germany), and their concentrations are shown in Table 7. We found a mixing ratio to show the properties of a good dichroic dye, and an injection phenomenon occurred when the concentration in the LC was 8 wt % or more.

Nematic LC (∆n = 0.1144, ∆ε = 5, MLC-7037, Darmstadt, Merck) was used for the polarized mode, and Chiral dopant, with left-handed properties of 360° chirality at 10 µm pitch, was mixed with nematic LC (TNI = 78.5 °C, ∆n = 0.108, E‖ = 16.5, E⊥ = 3.8, JNC Co., Tokyo, Japan) for the non-polarized mode at a ratio of 2.566 wt %, using GH effect. 

**Figure 8:**
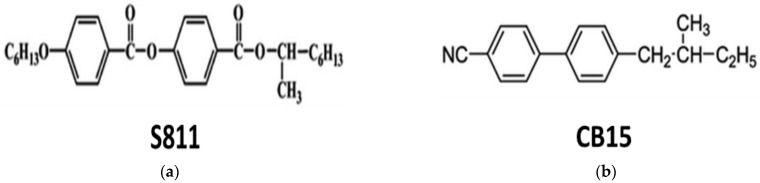
Chemical structures of two chiral dopants: (**a**) S811, (**b**) CB15.

Chirality has a significant impact on physical qualities of the physical system, such as molecular assemblies and crystal formation. More specifically, it is possible to induce a blue phase (BP) that is a factor in the optical isotropy, fast response time and high contrast ratio characteristics of LC. If the helical twisting power (HTP) of chiral dopant is larger, BPs can be induced with a small amount of dopant [27]. As shown in Figure 8, we prepared two chiral dopants for the fabrication of Ch-LC cell, i.e., 4-[1-methylheptyloxy]carbonylpheny]-4-(hexyloxy)benzoate (S811, Merck, HTP: 6.7 µm^−1^) and 4-cyano-4′-(2-methylbutyl)-biphenyl (CB15, Merck, HTP: 10.3 µm^−1^). There is a phase sequence Cr 4℃ N* 544℃ Iso in CB15, and it exhibits right-handed helix. A phase sequence in S811 is Cr 47.8℃ Iso, and S811 exhibits left-handed helix. We used S811 with higher HTP for Ch-LC fabrication.

**Figure 9:**
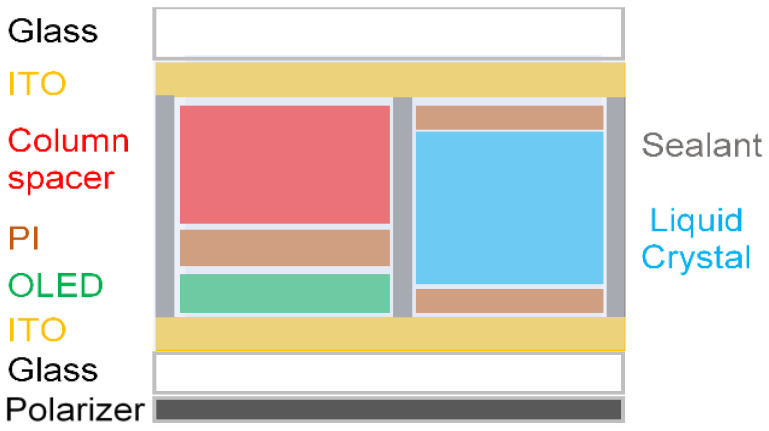
Experimental cell preparation for the proposed transparent display and this structure will apply only to the polarized mode.

For each mode used as a LC shutter, response time, voltage-transmittance of black and transparent, and contrast ratio were measured to optimize the fabricating conditions, according to electro-optical characteristics. Then the process conditions for fabricating the proposed transparent OLED display were optimized. To experiment the proposed polarized mode and non-polarized mode transparent OLED display, we have fabricated the transparent display panel consisting of an OLED cell for the image part and a LC cell for the window shutter on the single glass substrate, as shown in Figure 9.

**Figure 10:**
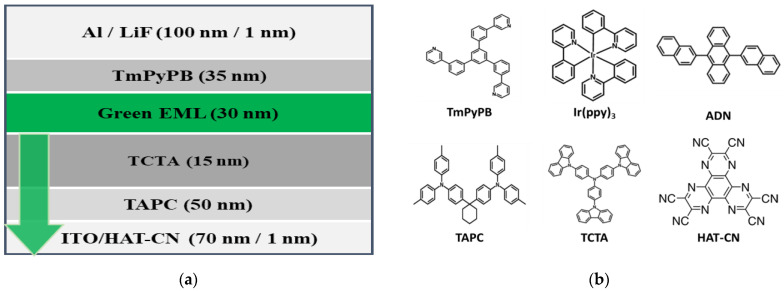
(**a**) OLED The OLED cell structure used, (**b**) Chemical structures of materials applied to OLED.

To fabricate the proposed transparent OLED display device, a green OLED device of bottom emission type was used. As shown in Figure 10a, the OLED was composed of Aluminum (Al) 100 nm; lithium fluoride (LiF) 1 nm; 1,3,5-Tri[(3-pyridyl)-phen-3-yl]benzene (TmPyPB) 35 nm; 9,10-di(naphtha-2-yl)anthracene (AND) with 8%-Tris(2-phenylpyridine)iridium(III) (Ir(ppy)_3_) 30 nm; 4,4′4”-Tris(carbazole-9-yl)triphenylamine (TCTA) 15 nm; di-[4-(*N,N*-di-p-tolyl-amino)-phenyl]cyclohexane (TAPC) 50 nm; dipyrazino [2,3-f:2′,3′-h]quinoxaline-2,3,6,7,10,11-hexacarbonitrile (HAT-CN) 1 nm; and indium tin oxide (ITO) 70 nm. The molecular structures of the consisting materials are presented in Figure 10b. A phosphorescent material was used as the material of the green emission layer; cathode was fabricated using 100% reflective electrode; and anode was fabricated using transparent electrode for light transmission.

In order to realize the proposed transparent OLED cell, an ITO electrode coated on the glass was simultaneously patterned for the OLED part and the LCD part in the cell. The ratio of the window part and the display image part was 1:1, and the cell gap of the LC was adjusted using a column spacer. In order to prevent leakage of the LC to OLED device area, a wall using sealant was fabricated, and we applied a bottom emission OLED type for the proposed hybrid-type transparent OLED display.

**Figure 11:**
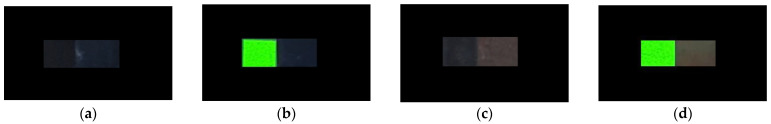
Fabrication and operation of polarized mode transparent OLED display is (**a**) when OLED and LCD are dark state, (**b**) when only OLED is bright state, (**c**) when only LCD is bright state, (**d**) when all is bright state.

**Figure 12:**
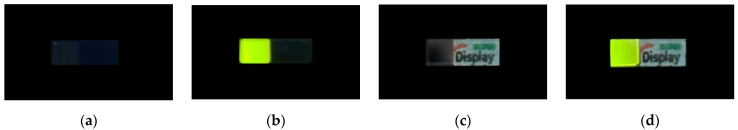
Fabrication and operation of non-polarized mode transparent OLED display is (**a**) when OLED and LCD are dark state, (**b**) when only OLED is bright state, (**c**) when only LCD is bright state, (**d**) when all is bright state.

First of all, the ITO was patterned using positive photoresist (AZ GXR-601, 14 cP, AZ Electronics Materials Co., Ltd., Tokyo, Japan) to fabricate the proposed cell. The bottom glass was patterned to realize the OLED part, and the top glass was patterned so that LC and OLED do not overlap. We have especially applied bottom emission type OLED structure because a cathode of the OLED reflects 100% of the emitted light so that it is never disturbed by the material on the top electrode when emitting light. Then polyimide (RN-1322, Nissan Chemical, Chouku, Japan) was coated on the bottom and top glass to coat the alignment layer, and photo rubbing was performed using ultraviolet ray (1200 mJ/cm^2^). And then, the column spacer was patterned using negative photoresist (AZ-5214, AZ Electronics Materials, Tokyo, Japan) on the bottom glass for controlling the thickness of the LC layer. Finally, the top and bottom glass were assembled, and then LC was injected into the empty cell. Figure 11 and Figure 12 show the bright and dark states of the fabricated polarized mode transparent OLED cell and non-polarized mode transparent OLED cell.

## 4. Conclusions

In general, a conventional transparent display using an OLED device with an empty window results in serious optical contrast because the light luminance in the dark state cannot be decreased due to the optically empty window, so that the dark state should be improved by blocking the windows when we need. In this paper, we could improve the optical contrast ratio of the transparent OLED cell by applying a LC switching mode to the empty window in the cell. We proposed two optical modes of the LC with/without a polarizer on the transparent window area. In the proposed transparent cell, we applied the Heilmeier structure with an ECB mode LC layer to the window area for the polarized mode transparent cell and also a Ch-LC layer to the window area for the non-polarized mode transparent cell. Two proposed LC modes for empty window have shown optical trade-off between the light luminance in the bright state and the contrast ratio. From the experiment, we observed that the measured contrast of the proposed structures has shown high optical improvement compared to the conventional transparent OLED cell without serious decay of the luminance in the bright state. Generally, the OLED cell would apply a polarizer to block the light reflection from outside, which depends on operating circumstances, so we believe that two proposed optical structures can be applied differently, depending on whether the transparent OLED cell applies to the polarizer or not.

## Figures and Tables

**Table 1:** Measured electro-optical characteristics of the polarized mode LC cell as a parameter of cell gap at 6.2 wt % of the dichroic dye concentration.

		On State		Off State		
Cell Gap (µm)	Response Time (ms)	Voltage (v)	Luminance (cd/m^2^)	Voltage (v)	Luminance (cd/m^2^)	ΔV (V)
1.6	30	6	331	0.4	112	5.6
2.6	37	7	296	1.3	66	5.7
3.2	44	9	293	1.5	62	7.5
5.6	57	11	244	2.1	37	8.9
7.2	68	11	202	2.2	30	6.8
8	77	14	193	2.8	28	11.2
9.2	83	14	136	3.3	16	10.7
10	90	14	120	3.5	13	10.5

**Table 2:** Measured electro-optical characteristics of the polarized mode LC cell as a parameter of the dye concentration at 5.6 µm of the cell gap.

		On State		Off State		
wt (%)	Response Time (ms)	Voltage (v)	Luminance (cd/m^2^)	Voltage (v)	Luminance (cd/m^2^)	ΔV (V)
3.4	50	9	304	1.5	59	7.5
5	51	9	274	1.8	45	7.2
6.2	57	11	244	2.2	37	8.8
7.4	60	11	229	2.4	29	8.6

**Table 3:** Measured electro-optical characteristics of the non-polarized mode LC cell as a parameter of the dye concentration at 10.0 µm of the cell gap.

		On State		Off State		
wt (%)	Response Time (ms)	Voltage (v)	Luminance (cd/m^2^)	Voltage (v)	Luminance (cd/m^2^)	ΔV (V)
3	178	6.0	654	5.2	138	0.8
4	234	6.6	530	5.6	100	1.0
5	278	8.4	442	6.2	72	2.2
6	315	8.8	359	6.2	45	2.6
7	334	8.6	266	6.4	32	2.2
8	389	8.8	110	6.4	18	2.4

**Table 4:** Light luminance and calculated contrast ratio of the polarized mode transparent OLED display as a parameter of cell gap at 6.2 wt % of the dichroic dye concentration. Light luminance in the OLED area was measured as 2162 cd/m^2^.

Cell Gap (μm)	Contrast Ratio
1.6	22:1
2.6	37:1
3.2	40:1
5.6	65:1
7.2	79:1
8	84:1
9.2	144:1
10	175:1

**Table 5:** Light luminance and calculated contrast ratio of the polarized mode transparent OLED display as a parameter of the dye concentration at 5.6 µm of the cell gap. Light luminance in the OLED area was measured as 2162 cd/m^2^.

wt %	Contrast Ratio
3.4	42:1
5	54:1
6.2	65:1
7.4	82:1

**Table 6:** Light luminance and calculated contrast ratio of the non-polarized mode transparent OLED display as a parameter of the dye concentration at 10.0 µm of the cell gap. Light luminance in the OLED area was measured as 4644 cd/m^2^.

wt %	Contrast Ratio
3	38:1
4	52:1
5	71:1
6	111:1
7	153:1
8	264:1

**Table 7:** Concentrations of blue, red, yellow and cyan for black dye development.

Concentration of Dichroic Dyes (wt %)	Liquid Crystal (g)	Type 1. Blue Dye (g)	Type 2. Red Dye (g)	Type 3. Yellow Dye (g)	Type 4. Cyan Dye (g)
3%	2.91	0.02727	0.02727	0.02727	0.0081
4%	2.88	0.03636	0.03636	0.03636	0.0109
5%	2.85	0.04545	0.04545	0.04545	0.0135
6%	2.82	0.05454	0.05454	0.05454	0.0163
7%	2.79	0.06363	0.06363	0.06363	0.0190
8%	2.76	0.07272	0.07272	0.07272	0.0218

## Data Availability

Not applicable.
